# Adiabatic Amplification of the Harmonic Oscillator Energy When the Frequency Passes through Zero

**DOI:** 10.3390/e25010002

**Published:** 2022-12-20

**Authors:** Viktor V. Dodonov, Alexandre V. Dodonov

**Affiliations:** 1 Institute of Physics, University of Brasilia, P.O. Box 04455, Brasilia 70919-970, DF, Brazil; 2 International Center of Physics, University of Brasilia, Brasilia 70910-900, DF, Brazil

**Keywords:** adiabatic invariants, Born–Fock theorem, power-law time-dependent frequency, Epstein–Eckart frequency dependence, mean energy, energy fluctuations, invariant squeezing

## Abstract

We study the evolution of the energy of a harmonic oscillator when its frequency slowly varies with time and passes through a zero value. We consider both the classical and quantum descriptions of the system. We show that after a single frequency passage through a zero value, the famous adiabatic invariant ratio of energy to frequency (which does not hold for a zero frequency) is reestablished again, but with the proportionality coefficient dependent on the initial state. The dependence on the initial state disappears after averaging over the phases of initial states with the same energy (in particular, for the initial vacuum, the Fock and thermal quantum states). In this case, the mean proportionality coefficient is always greater than unity. The concrete value of the mean proportionality coefficient depends on the power index of the frequency dependence on a time near the zero point. In particular, the mean energy triplicates if the frequency tends to zero linearly. If the frequency attains zero more than once, the adiabatic proportionality coefficient strongly depends on the lengths of time intervals between zero points, so that the mean energy behavior becomes quasi-stochastic after many passages through a zero value. The original Born–Fock theorem does not work after the frequency passes through zero. However, its generalization is found: the initial Fock state becomes a wide superposition of many Fock states, whose weights do not depend on time in the new adiabatic regime. When the mean energy triplicates, the initial Nth Fock state becomes a superposition of, roughly speaking, 6N states, distributed nonuniformly. The initial vacuum and low-order Fock states become squeezed, as well as the initial thermal states with low values of the mean energy.

## 1. Introduction

One of many brilliant results of classical and quantum mechanics is the existence of *adiabatic invariants* in the case where parameters of a system vary slowly with time. The simplest invariant is the ratio of the energy of a harmonic oscillator E(t) to its time-dependent frequency ω(t) [1]:

(1) $$E\left(t\right)/\omega \left(t\right)=const\;\mathrm{if}\;|\dot{\omega }|/\omega^{2}\left(t\right)\ll 1.$$

Then, suppose that the frequency returns to its initial value after some slow variations. What is the final energy of the oscillator? According to Equation (1), the answer is obvious: the final energy coincides with the initial one. However, there exists a remarkable exclusion from this result, when the frequency passes through a zero value in the process of evolution, so that the condition of validity of Equation (1) is obviously broken for any rate of the evolution. The goal of our paper is to study the dependence of the final energy on the shape of the time-dependent frequency, when this frequency slowly passes through a zero value. We perform analytic calculations for the quantum oscillator and numeric calculations for the classical oscillator. Note that various aspects of the harmonic oscillator evolution in the adiabatic regime have been studied by many authors for decades (see, e.g., papers [2,3,4,5,6,7,8]). However, the situation when the frequency passes slowly through a zero value have not been considered in the known publications. An oscillator whose frequency exponentially goes adiabatically and asymptotically to zero has been considered, e.g., in papers [9,10]. However, we are interested in the case when the frequency passes through zero and returns to its initial value.

The structure of the paper is as follows. In Section 2, we describe the results of the numeric solutions of the classical equations of motion for two kinds of time dependence of the frequency: $\omega^{2}\left(t\right)∼{\left|t\right|}^{n}$ and $\omega^{2}\left(t\right)∼{\left|\tanh\left(at\right)\right|}^{n}$. Several figures show the differences between the cases of fast and slow frequency variations, when the frequency does not attain a zero value and when it passes through a zero value. In Section 3, we present general formulas describing the evolution of the mean oscillator energy in the quantum case, including adiabatic regimes without and with a zero-crossing frequency value. Exact solutions for the power profile of the frequency are derived and analyzed in Section 4. Transition rules in the case of a single frequency passage through zero are obtained in Section 5. An example of tanh-like frequency functions is considered in Section 6. Double transitions of the frequency through zero values are studied in Section 7 in the quantum and classical cases. Section 8, Section 9 and Section 10 are devoted to the energy fluctuations, the violation and generalization of the Born–Fock theorem and the appearance of squeezing, respectively. Section 11 contains a discussion of the main results.

## 2. Evolution of the Oscillator Energy in the Classical Case

The basic equation has the form

(2) $$\ddot{x}+\omega^{2}\left(t\right)x=0.$$

We consider a special case when the time-dependent frequency can be written as $\omega^{2}\left(t\right)=\omega_{0}^{2}f\left(t/\tau \right)$, where f(t/τ) is a non-negative function with the properties

(3) f(−1)=1,f(0)=0,τ>0.

We assume that $\omega^{2}\left(t\right)=\omega_{0}^{2}=const$ for t≤−τ. Since we are interested mainly in the evolution of the energy at t>−τ, we consider a one-parameter family of classical trajectories with the same initial energy $E_{0}$. Then, assuming the particle mass m=1, it is convenient to use the initial coordinate $x_{0}$ and initial velocity ${\dot{x}}_{0}$ in the form

$$x_{0}=\cos\left(\varphi \right)\sqrt{2E_{0}}/\omega_{0},\;{\dot{x}}_{0}=\sin\left(\varphi \right)\sqrt{2E_{0}},\;0\leq \varphi <2\pi .$$

To solve Equation (2) numerically, we introduce dimensionless variables $X=\omega_{0}x/\sqrt{2E_{0}}$ and T=t/τ, arriving at the equation for T≥−1

(4) $$d^{2}X/dT^{2}+G^{2}f\left(T\right)X=0,\;G=\omega_{0}\tau ,$$

with the initial conditions

(5) $${X\left(-1\right)=\cos\left(\varphi \right),\;dX/dT|}_{T=-1}=G\sin\left(\varphi \right).$$

Then, the dimensionless energy ratio $R=E\left(t\right)/E_{0}$ depends on the dimensionless time *T* and two parameters, *G* and φ:

(6) $$R\left(T;G;\varphi \right)=f\left(T\right)X^{2}\left(T\right)+G^{-2}{\left(dX/dT\right)}^{2},$$

where the derivative dX/dT must be taken at instant *T*. The existence of the adiabatic invariant (1) implies that $R\left(T;G\right)=\sqrt{f(T)}$ for T<0 and big enough values of parameter *G*, *independently on the values of parameter φ*. In the following subsections, we study what can happen if T>0, for different families of functions f(T).

### 2.1. A Power Profile of the Frequency

Our first example is the power profile $f\left(T\right)={\left|T\right|}^{n}$ with n>0. We solved Equation (4) numerically and calculated the dimensionless ratio

$$R_{n}\left(T;G;\varphi \right)={\left|T\right|}^{n}X^{2}\left(T\right)+G^{-2}{\left(dX/dT\right)}^{2}.$$

The adiabatic ratio must equal $R_{n}\left(T;G\right)={\left|T\right|}^{n/2}$. In Figure 1 and Figure 2, we show $R_{n}$ as function of φ for the fixed values T=−1/2, n=1/2, n=2 and n=4, comparing different behaviors when G=1 (no adiabaticity) and G=1000. Figure 1 shows a strong dependence of the energy on the phase φ in the nonadiabatic regime. However, this dependence becomes negligible in Figure 2, which shows that the mean value of $R_{n}$ is, indeed, ${\left|T\right|}^{n/2}$.

However, the situation becomes quite different for T≥0: the dependence of $R_{n}$ on φ does not disappear even for very big values of parameter *G*. This is shown in Figure 3 for $R_{n}\left(1;1000;\varphi \right)$, with n=1/2, n=2 and n=4. It is important to pay attention to the different vertical scales in the different plots. Strong oscillations are observed. The mean value of these oscillations depends on index *n*.

### 2.2. A Tanh-like Profile of the Frequency

The second example is the profile $f\left(T\right)={\left|\tanh\left(aT\right)/\tanh\left(-a\right)\right|}^{n}$ with n>0 and a=5, which describes a softer transition from the constant frequency to a time-dependent one. The plots in this case turn out very similar to those of the preceding section. We show one of them in Figure 4, for the value T=1 (when the final frequency coincides with the initial one). While the lines R(φ) are shifted with respect to each other in the cases of $f\left(T\right)={\left|T\right|}^{n}$ and $f\left(T\right)={\left|\tanh\left(aT\right)/\tanh\left(-a\right)\right|}^{n}$, the maximal and minimal values coincide. Moreover, the average values of the final energy as functions of index *n* turn out practically identical for two families of frequency profiles, as shown in Figure 5. This coincidence is explained in the following sections.

## 3. Evolution of the Mean Oscillator Energy in the Quantum Case

In the quantum case, one has to solve the time-dependent Schrödinger equation and use the solutions to calculate various mean values, in particular, those contributing to the mean energy. However, a simpler way is to use the Ehrenfest equations for the mean values, which are immediate consequences of the Schrödinger equation. It was shown in the seminal papers by Husimi [11], Popov and Perelomov [12], Lewis and Riesenfeld [13] and Malkin, Man’ko and Trifonov [14] that the solutions of the Schrödinger and Ehrenfest equations for the harmonic oscillator with an arbitrary time-dependent frequency depend on complex functions ε(t) and $\varepsilon^{*}\left(t\right)$, satisfying Equation (2) and the initial conditions

(7) $$\varepsilon \left(-\tau \right)=\omega_{0}^{-1/2},\;\dot{\varepsilon }\left(-\tau \right)=i\omega_{0}^{1/2}.$$

The Wronskian identity for the solutions ε(t) and $\varepsilon^{*}\left(t\right)$ has the form

(8) $$\dot{\varepsilon }\varepsilon^{*}-{\dot{\varepsilon }}^{*}\varepsilon =2i.$$

Then, we can write at t≥τ

(9) x(t)=x0ω0Re[ε(t)]+p0ω0Im[ε(t)],p(t)=x0ω0Re[ε˙(t)]+p0ω0Im[ε˙(t)].

Equation (9) holds both for the classical and quantum particles (in the Heisenberg representation in the latter case). Its immediate consequences are the following formulas for the second-order moments of the canonical operators for t≥−τ:

(10) 〈x2〉t=〈x2〉−τω0Re[ε(t)]2+〈p2〉−τω0Im[ε(t)]2+〈xp+px〉−τRe[ε(t)]Im[ε(t)],

(11) 〈p2〉t=〈x2〉−τω0Re[ε˙(t)]2+〈p2〉−τω0Im[ε˙(t)]2+〈xp+px〉−τRe[ε˙(t)]Im[ε˙(t)].

The time-dependent mean energy is given by the formula

(12) $$E\left(t\right)=\frac{1}{2}\left[{\langle p^{2}\rangle }_{t}+\omega^{2}\left(t\right){\langle x^{2}\rangle }_{t}\right].$$

It is worth remembering that for systems with *quadratic* Hamiltonians with respect to *x* and *p*, the dynamics of the first-order mean values 〈x〉 and 〈p〉 are *totally independent* from the dynamics of the variances $\sigma_{x}=\langle x^{2}\rangle -{\langle x\rangle }^{2}$, $\sigma_{p}=\langle p^{2}\rangle -{\langle p\rangle }^{2}$ and $\sigma_{xp}=\langle xp+px\rangle /2-\langle x\rangle \langle p\rangle$. This means that the equations of the same form as (10) and (11) exist for the sets $\left({\langle x\rangle }^{2},{\langle p\rangle }^{2},\langle x\rangle \langle p\rangle \right)$ and $\left(\sigma_{x},\sigma_{p},\sigma_{xp}\right)$.

The adiabatic (quasi-classical) approximate complex solution to Equation (2), satisfying the initial conditions (7), has the form

(13) $$\varepsilon \left(t\right)\approx {\left[\omega \left(t\right)\right]}^{-1/2}e^{i\phi_{\tau }\left(t\right)},\;\dot{\varepsilon }\left(t\right)\approx i{\left[\omega \left(t\right)\right]}^{1/2}e^{i\phi_{\tau }\left(t\right)},\;\phi_{\tau }\left(t\right)=\int_{-\tau }^{t}\omega \left(z\right)dz.$$

Putting the solution (13) in the Equations (10)–(12), we arrive immediately at the adiabatic invariant

(14) $$E\left(t\right)/\omega \left(t\right)=E\left(-\tau \right)/\omega_{0},$$

*for arbitrary initial values* at t=−τ. However, the solution (13) obviously loses its sense if ω(t)=0 at some time instant $t_{*}$ (taken as t=0 in our paper). Nonetheless, when the frequency slowly passes through a zero value and slowly becomes not too small, the conditions of the quasi-classical approximation are reestablished again. Hence, the solution for t>0 can be written (outside some interval near t=0) in the most general quasi-classical form as follows,

(15) $$\varepsilon \left(t\right)\approx {\left[\omega \left(t\right)\right]}^{-1/2}\left[u_{+}e^{i\phi (t)}+u_{-}e^{-i\phi (t)}\right],\;\dot{\varepsilon }\left(t\right)\approx i{\left[\omega \left(t\right)\right]}^{1/2}\left[u_{+}e^{i\phi (t)}-u_{-}e^{-i\phi (t)}\right],$$

where

(16) $$\phi \left(t\right)=\int_{t_{*}}^{t}\omega \left(z\right)dz,\;d\phi \left(t\right)/dt=\omega \left(t\right).$$

Constant complex coefficients $u_{\pm }$ must obey the condition

(17) $$|u_{+}{|}^{2}-{\left|u_{-}\right|}^{2}=1,$$

which is the consequence of Equation (8). Then, Equation (12) assumes the form

(18) $$\frac{{\langle E\rangle }_{t}}{{\langle E\rangle }_{-\tau }}=\frac{\omega (t)}{\omega_{0}}\left(\beta +\Delta \beta \right),$$

where

(19) $$\beta =|u_{+}{|}^{2}+|u_{-}{|}^{2}=1+2{\left|u_{-}\right|}^{2},$$

(20) $$\Delta \beta =\left\{\left[\omega_{0}^{2}{\langle x^{2}\rangle }_{-\tau }-{\langle p^{2}\rangle }_{-\tau }\right]Re\left(u_{+}u_{-}\right)+\omega_{0}{\langle xp+px\rangle }_{-\tau }Im\left(u_{+}u_{-}\right)\right\}/{\langle E\rangle }_{-\tau }.$$

Equation (18) can be interpreted as a generalized adiabatic formula for the energy after the frequency passes *slowly* through a zero value. It shows that the quantum mechanical mean energy is proportional to the instant frequency ω(t) in the adiabatic regime. However, the proportionality coefficient strongly depends on the initial conditions in the most general case. This is in agreement with the classical results shown in Figure 3 and Figure 4. For this reason, we concentrate hereafter on the important special case when

(21) $${\langle p^{2}\rangle }_{-\tau }=\omega_{0}^{2}{\langle x^{2}\rangle }_{-\tau },\;{\langle xp+px\rangle }_{-\tau }=0.$$

It includes the vacuum, thermal and Fock initial quantum states. Then, Δβ=0. In addition, many formulas can be simplified:

(22) $${\langle x^{2}\rangle }_{t}=\omega_{0}{\langle x^{2}\rangle }_{-\tau }|\varepsilon \left(t\right){|}^{2},\;{\langle p^{2}\rangle }_{t}={\langle p^{2}\rangle }_{-\tau }{\left|\dot{\varepsilon }\left(t\right)\right|}^{2}/\omega_{0},$$

(23) $${\langle E\rangle }_{t}=\frac{{\langle E\rangle }_{-\tau }}{2\omega_{0}}\left[\omega^{2}{\left(t\right)|\varepsilon \left(t\right)|}^{2}+{\left|\dot{\varepsilon }\left(t\right)\right|}^{2}\right].$$

In principle, the choice of the initial point of integration in Equation (16), defining the phase function ϕ(t) in Equation (15) can be arbitrary, since it influences the phases of coefficients $u_{\pm }$ only. However, the point $t_{*}=0$ is distinguished in our problem, because $\omega (t_{*})=0$. Therefore, we assume hereafter that $t_{*}=0$ in the definition of the phase ϕ(t) (16).

Note that after Lewis and Riesenfeld’s paper [13], many authors working on various problems related to the harmonic oscillator with a time-dependent frequency did not use as a starting point linear Equation (2) but its nonlinear analog (known under the name “Ermakov equation”)

(24) $$\ddot{\rho }+\omega^{2}\left(t\right)\rho =\rho^{-3},\;\rho \left(t\right)\equiv \left|\varepsilon \left(t\right)\right|,$$

which follows from (2) if one writes ε=ρexp(iχ) and takes into account the condition (8). Then, one can rewrite Equation (23) as follows,

(25) $${\langle E\rangle }_{t}=\frac{{\langle E\rangle }_{-\tau }}{2\omega_{0}}\left\{\omega^{2}\left(t\right)\rho^{2}\left(t\right)+{\left[\dot{\rho }\left(t\right)\right]}^{2}+{\left[\rho \left(t\right)\right]}^{-2}\right\}\geq \frac{{\langle E\rangle }_{-\tau }}{2\omega_{0}}\left\{2|\omega \left(t\right)|+{\left[\dot{\rho }\left(t\right)\right]}^{2}\right\}.$$

Consequently, the mean energy always *increases* when the frequency returns to its initial value, unless the time derivative $\dot{\rho }\left(t\right)$ is negligibly small, i.e., if $u_{-}\neq 0$ (under the condition (21)). Many references on the subjects related to the Ermakov equation can be found, e.g., in the review [15] and a recent paper [16]. However, we prefer to use linear equation (2), because the key terms that help us solve the adiabatic problem are the coefficients $u_{\pm }$ in the asymptotic formula (15).

However, how to find these constant coefficients $u_{\pm }$? The numeric results of Section 2 (especially Figure 5) indicate that the answer depends on the exponent *n* in the form of the frequency transition through zero: $\omega^{2}\left(t\right)∼{\left|t\right|}^{n}$ when t→0 (assuming that ω(t)=0 at t=0). It is remarkable that the explicit dependence of $|u_{-}{|}^{2}$ on the index *n* can be found analytically, as shown in the next section.

## 4. Exact Solutions for the Power Profile of the Frequency

It is known that Equation (2) with the time-dependent frequency $\omega^{2}\left(t\right)=\omega_{0}^{2}{\left|t/\tau \right|}^{n}$ can be reduced to the Bessel equation

(26) $$\frac{d^{2}Z}{dy^{2}}+\frac{1}{y}\frac{dZ}{dy}+\left(1-\frac{\nu^{2}}{y^{2}}\right)Z=0$$

for t>0 (see, e.g., papers [17,18]). The same can be done for t<0, as soon as the initial equation is invariant with respect to the time reflection t→−t. One can verify that Equation (2) goes to (26) with the aid of the following transformations:

(27) $$x\left(t\right)=\sqrt{\left|t\right|}\;Z\left[y\left(t\right)\right],\;\nu =\frac{1}{n+2},\;y\left(t\right)=g{\left|\frac{t}{\tau }\right|}^{\gamma },\;\gamma =\frac{1}{2\nu },\;g=2G\nu ,\;G=\omega_{0}\tau .$$

Hence, the function ε(t) can be written as a superposition of the Bessel functions $J_{\nu }\left(y\right)$ and $J_{-\nu }\left(y\right)$, although with different coefficients in the regions of t<0 and t>0:

(28) $$\varepsilon \left(t\right)=\sqrt{\left|t\right|}\times \left\{\begin{array}{cc} \left\{A_{-}J_{\nu }\left[y\left(t\right)\right]+B_{-}J_{-\nu }\left[y\left(t\right)\right]\right\}, & t<0\\ \left\{A_{+}J_{\nu }\left[y\left(t\right)\right]+B_{+}J_{-\nu }\left[y\left(t\right)\right]\right\}, & t>0 \end{array}\right..$$

Constant complex coefficients $A_{-}$ and $B_{-}$ can be found from the initial conditions (7). Remembering that d|t|/dt=−1 for t<0, one obtains the following equations:

$$A_{-}J_{\nu }\left(g\right)+B_{-}J_{-\nu }\left(g\right)=1/\sqrt{G},$$

$$A_{-}J_{\nu }^{\prime }\left(g\right)+B_{-}J_{-\nu }^{\prime }\left(g\right)=-\;\frac{1}{\sqrt{G}}\left(i+\frac{1}{2G}\right),$$

where $J_{\pm \nu }^{\prime }\left(z\right)$ means the derivative of the Bessel function $J_{\pm \nu }\left(z\right)$ with respect to its argument *z*. Using the known Wronskian [19,20]

$$J_{\nu }\left(z\right)J_{-\nu }^{\prime }\left(z\right)-J_{-\nu }\left(z\right)J_{\nu }^{\prime }\left(z\right)=-2\sin\left(\nu \pi \right)/\left(z\pi \right),$$

we obtain the following expressions:

$$A_{-}=-\;\frac{\nu \pi \sqrt{G}}{\sin(\nu \pi )}\left[J_{-\nu }^{\prime }\left(g\right)+\left(i+\frac{1}{2G}\right)J_{-\nu }\left(g\right)\right],$$

$$B_{-}=\frac{\nu \pi \sqrt{G}}{\sin(\nu \pi )}\left[\left(i+\frac{1}{2G}\right)J_{\nu }\left(g\right)+J_{\nu }^{\prime }\left(g\right)\right].$$

Using the known identities (see, e.g., formulas 7.2 (54) and 7.2 (55) in [20])

(29) $$J_{\nu }\left(z\right)\pm \frac{z}{\nu }J_{\nu }^{\prime }\left(z\right)=\frac{z}{\nu }J_{\nu \mp 1}\left(z\right),$$

we can simplify formulas for the coefficients $A_{-}$ and $B_{-}$:

(30) $$A_{-}=\frac{\nu \pi \sqrt{G}}{\sin(\nu \pi )}\left[J_{1-\nu }\left(g\right)-iJ_{-\nu }\left(g\right)\right],\;B_{-}=\frac{\nu \pi \sqrt{G}}{\sin(\nu \pi )}\left[iJ_{\nu }\left(g\right)+J_{\nu -1}\left(g\right)\right].$$

The time derivative of function (28) at t<0 (when d|t|/dt=−1) can be written with the aid of identities (29) as follows:

(31) $$d\varepsilon /dt=\frac{y}{2\nu \sqrt{\left|t\right|}}\left[B_{-}J_{1-\nu }\left(y\right)-A_{-}J_{\nu -1}\left(y\right)\right],\;t\leq 0.$$

On the other hand,

(32) $$d\varepsilon /dt=\frac{y}{2\nu \sqrt{t}}\left[A_{+}J_{\nu -1}\left(y\right)-B_{+}J_{1-\nu }\left(y\right)\right],\;t\geq 0.$$

Using the leading term of the Bessel function $J_{p}\left(z\right)=z^{p}/\left[2^{p}\Gamma \left(p+1\right)\right]$ at z→0, one can see that $\sqrt{\left|t\right|}J_{\nu }\left(y\right)\to 0$ when t→0, while the product $\sqrt{\left|t\right|}J_{-\nu }\left(y\right)$ tends to a finite value in this limit. Consequently, the continuity of function ε(t) at t=0 implies the condition $B_{+}=B_{-}$. On the other hand, $yJ_{1-\nu }\left(y\right)/\sqrt{\left|t\right|}\to 0$ at t→0, while the product $yJ_{\nu -1}\left(y\right)/\sqrt{\left|t\right|}$ tends to a finite value in this limit. Hence, the continuity of derivative dε/dt at t=0 can be guaranteed under the condition $A_{+}=-A_{-}$. Then, one can verify that the Wronskian identity (8) is satisfied identically, both for t≤0 and t≥0, in view of the identity [20]

(33) $$J_{\nu }\left(z\right)J_{1-\nu }\left(z\right)+J_{-\nu }\left(z\right)J_{\nu -1}\left(z\right)=2\sin\left(\nu \pi \right)/\left(z\pi \right).$$

Using Equations (28) and (30), one can write down formula (23) for the mean energy ratio R(t)=E(t)/E(−τ) as follows:

(34) $$\left.\begin{array}{c} R(t<0)\\ R(t>0) \end{array}\right\}=\frac{1}{8}{\left[\frac{g\pi }{\sin(\nu \pi )}\right]}^{2}{\left|\frac{t}{\tau }\right|}^{n+1}\left[K_{-}\left(g\right)K_{+}\left(y\right)+K_{+}\left(g\right)K_{-}\left(y\right)\mp 2K_{0}\left(g\right)K_{0}\left(y\right)\right],$$

where

$$K_{+}\left(z\right)=J_{\nu }^{2}\left(z\right)+J_{\nu -1}^{2}\left(z\right),\;K_{-}\left(z\right)=J_{-\nu }^{2}\left(z\right)+J_{1-\nu }^{2}\left(z\right),\;K_{0}\left(z\right)=J_{\nu -1}\left(z\right)J_{1-\nu }\left(z\right)-J_{\nu }\left(z\right)J_{-\nu }\left(z\right).$$

Figure 6 shows the function R(b), where −1≤b≡t/τ≤1, for ν=1/4 (i.e., n=2 and $y=gb^{2}$) and three values of parameter g=0.1,1.0,10.

Using the known formulas

(35) $$J_{\nu }\left(y\right)\approx {\left(y/2\right)}^{\nu }/\Gamma \left(\nu +1\right),\;y\ll 1,$$

(36) Γ(z)Γ(1−z)=π/sin(πz),

one can see that R(b) is totally symmetric in the limit g→0 (an instantaneous frequency jump through a zero value), when $R\left(b\right)=\left(1+{\left|b\right|}^{n}\right)/2$. However, the symmetry is broken for not very small values of parameter *g*. The known asymptotic formula for the Bessel functions of large arguments [19,20],

(37) $$J_{\nu }\left(z\right)∼\sqrt{\frac{2}{\pi z}}\cos\left(z-\frac{\nu \pi }{2}-\frac{\pi }{4}\right),$$

results in the following simple expressions for z≫1:

$$K_{\pm }\left(z\right)=\frac{2}{\pi z},\;K_{0}\left(z\right)=\frac{2\cos(\nu \pi )}{\pi z}.$$

Hence, in the adiabatic limit (g≫1 and y≫1), we obtain

$$\left.\begin{array}{c} R(t<0)\\ R(t>0) \end{array}\right\}=\frac{\omega \left(t\right)\left[1\mp \cos^{2}\left(\nu \pi \right)\right]}{\omega_{0}\sin^{2}\left(\nu \pi \right)}.$$

If t<0, we arrive exactly at the adiabatic formula (1) for any value of the power *n*. On the other hand, if t>0 (i.e., after the frequency has passed through a zero value), we see again the linear proportionality

(38) $$R\left(t\right)=\beta \omega \left(t\right)/\omega_{0},\;\beta =\frac{1+\cos^{2}\left(\nu \pi \right)}{\sin^{2}\left(\nu \pi \right)},\;\nu =\frac{1}{n+2}.$$

The proportionality coefficient β depends on parameter *n*. For example, β=5/3 for n=1, β=3 for n=2 and β=7 for n=4, in accordance with Figure 5. If n≪1, then β≈1, while $\beta \approx 2{\left(n/\pi \right)}^{2}$ for n≫1. To see the limitations on the validity of the adiabatic approximation g≫1, we plot in Figure 7 the ratio ρ≡R(τ),

(39) $$\rho =\frac{1}{4}{\left[\frac{g\pi }{\sin(\nu \pi )}\right]}^{2}\left[K_{-}\left(g\right)K_{+}\left(g\right)+K_{0}^{2}\left(g\right)\right],$$

as a function of *g* for ν=1/3,1/4,1/6. We see that the generalized adiabatic approximation (38) has an accuracy better than 1% for g>100.

In view of formula (35), the only nonzero contribution to the right-hand side of Equation (34) at t→0 is given by the function $K_{+}\left(y\right)\approx J_{\nu -1}^{2}\left(y\right)\approx {\left[{\left(y/2\right)}^{\nu -1}/\Gamma \left(\nu \right)\right]}^{2}∼{\left|t\right|}^{-(n+1)}$, whereas the contributions of $K_{-}\left(y\right)∼{\left|t\right|}^{-1}$ and $K_{0}\left(y\right)∼{\left|t\right|}^{0}$ are eliminated by the term ${\left|t\right|}^{\left(n+1\right)}$. Hence,

(40) $$R\left(t=0\right)=\frac{\pi g^{2\nu -1}}{{\left[2^{\nu }\Gamma \left(\nu \right)\sin\left(\pi \nu \right)\right]}^{2}},\;g\gg 1.$$

This means, in particular, that the adiabatic formula (38) holds under the condition $\omega \left(t\right)/\omega_{0}\gg g^{2\nu -1}$ (provided $g=2\nu \omega_{0}\tau \gg 1$).

## 5. Transition Rules for Adiabatic Coefficients after Frequency Passes through Zero Value

Equation (38) means that parameter $|u_{-}|$ (determining the adiabatic evolution of the mean energy after the frequency passes through a zero value according to formula (18)) has the following form:

(41) $$|u_{-}|=\cot\left(\nu \pi \right)=\cot\left(\frac{\pi }{n+2}\right).$$

This formula can be derived directly from Equation (28). If g≫1, Equation (30) assumes the following asymptotic form:

(42) $$A_{-}\approx \frac{\sqrt{\nu \pi }}{\sin(\nu \pi )}\exp\left[i\left(g+\frac{\nu \pi }{2}-\frac{3\pi }{4}\right)\right],\;B_{-}\approx \frac{\sqrt{\nu \pi }}{\sin(\nu \pi )}\exp\left[i\left(g-\frac{\nu \pi }{2}+\frac{\pi }{4}\right)\right]$$

Then, Equation (28) results in the following expressions for y≫1:

(43) $$\varepsilon \left(t<0\right)\approx {\left[\omega \left(t\right)\right]}^{-1/2}e^{i(g-y)},\;\varepsilon \left(t>0\right)\approx \frac{e^{ig}}{{\left[\omega \left(t\right)\right]}^{1/2}\sin\left(\nu \pi \right)}\left[e^{iy}+i\cos\left(\nu \pi \right)e^{-iy}\right].$$

On the other hand, calculating the phase ϕ(t) according to the definition (16), we find ϕ=−y for t<0 and ϕ=y for t>0. Hence, omitting the common phase term $e^{ig}$ in Equation (43) and comparing this equation with (13) and (15), we obtain the following expressions for the coefficients $u_{\pm }$:

(44) $$u_{+}={\left[\sin\left(\nu \pi \right)\right]}^{-1},\;u_{-}=i\cot\left(\nu \pi \right).$$

They satisfy exactly the identity (17) and result in formula (41). Note that coefficient $u_{+}$ given by Equation (44) is real. However, probably, the reality of this coefficient is due to the specific exact power shape of function ω(t) considered in this section. For other functions ω(t) with a similar behavior when ω→0, this coefficient can be complex, although with the same absolute value. An example is given in the next section. However, the formulas for the absolute values $|u_{\pm }|$,

(45) $$|u_{+}{|=\left[\sin\left(\nu \pi \right)\right]}^{-1},\;\left|u_{-}\right|=\cot\left(\nu \pi \right),$$

seem to be universal after a single frequency passage through zero.

## 6. Exact Solution for the Tanh Profile of the Frequency

An interesting example of exact solutions corresponds to the time-dependent frequency (a special case of the family of Epstein–Eckart profiles [21,22])

(46) $$\omega^{2}\left(t\right)=\omega_{0}^{2}\tanh^{2}\left(\kappa t/2\right),\;-\infty <t<\infty ,\;\kappa >0.$$

In this case, solutions to Equation (2) can be written in terms of the Gauss hypergeometric function

$$F\left(a,b;c;x\right)=\sum_{n=1}^{\infty }\frac{{\left(a\right)}_{n}{\left(b\right)}_{n}x^{n}}{{\left(c\right)}_{n}n!},$$

satisfying the equation

(47) $$x\left(1-x\right)F^{\prime \prime }+\left(c-\left(a+b+1\right)x\right)F^{\prime }-abF=0.$$

The first step to come to Equation (47) is to introduce the new variable ξ=tanh(κt/2). Then, Equation (2) takes the form

(48) $${\left(1-\xi^{2}\right)}^{2}\frac{d^{2}x}{d\xi^{2}}-2\xi \left(1-\xi^{2}\right)\frac{dx}{d\xi }+4{\tilde{\omega }}_{0}^{2}\xi^{2}x=0,\;{\tilde{\omega }}_{0}\equiv \omega_{0}/\kappa .$$

We wish to arrive to the function F(a,b;c;y) with y=(1+ξ)/2. In such a case, y=0 when t=−∞, so that the initial condition (7) can be easily satisfied, as soon as F(a,b;c;0)=1. On the other hand, there are many relations for the function F(a,b;c;1), which arises when t→∞. Then, the asymptotics of function ε(t) can be easily found. Therefore, looking for the solution in the form $x\left(t\right)={\left[y\left(1-y\right)\right]}^{{}^{\alpha }}f\left(y\right)$, we obtain the equation

(49) $$y^{2}{\left(1-y\right)}^{2}f^{\prime \prime }+y\left(1-y\right)\left(1-2y\right)\left(1+2\alpha \right)f^{\prime }+\left[{\left(2y-1\right)}^{2}\left(\alpha^{2}+{\tilde{\omega }}_{0}^{2}\right)-2\alpha y\left(1-y\right)\right]f=0.$$

Consequently, choosing $\alpha =i{\tilde{\omega }}_{0}$, we arrive at the solution

(50) $$\varepsilon \left(t\right)=\omega_{0}^{-1/2}{\left[y\left(1-y\right)\right]}^{i{\tilde{\omega }}_{0}}F\left(a_{+},a_{-};c;y\right)=\omega_{0}^{-1/2}{\left[2\cosh\left(\kappa t/2\right)\right]}^{-2i{\tilde{\omega }}_{0}}F\left(a_{+},a_{-};c;y\right),$$

where

(51) $$y=\frac{1}{2}\left[1+\tanh\left(\kappa t/2\right)\right]={\left(1+e^{-\kappa t}\right)}^{-1},$$

(52) $$a_{\pm }=\frac{1}{2}+2i{\tilde{\omega }}_{0}\pm r,\;r=\frac{1}{2}\sqrt{1-16{\tilde{\omega }}_{0}^{2}},\;c=1+2i{\tilde{\omega }}_{0}.$$

If t→−∞, function (50) goes to $\varepsilon \left(t\right)=\omega_{0}^{-1/2}\exp\left(i\omega_{0}t\right)$. If t→∞ (and y→1), we can use the analytic continuation of the hypergeometric function, given, e.g., by formula 2.10(1) from [20],

(53) $$\begin{array}{ccc} F(a,b;c;y) & = & \frac{\Gamma (c)\Gamma (c-a-b)}{\Gamma (c-a)\Gamma (c-b)}F\left(a,b;a+b+1-c;1-y\right)\\ & & +\frac{\Gamma (c)\Gamma (a+b-c)}{\Gamma (a)\Gamma (b)}{\left(1-y\right)}^{c-a-b}F\left(c-a,c-b;c+1-a-b;1-y\right). \end{array}$$

Then, function (50) assumes the asymptotic form

(54) $$\varepsilon \left(t\right)=\omega_{0}^{-1/2}\left[v_{+}e^{i\omega_{0}t}+v_{-}e^{-i\omega_{0}t}\right],$$

(55) $$v_{+}=\frac{\Gamma \left(1+2i{\tilde{\omega }}_{0}\right)\Gamma \left(2i{\tilde{\omega }}_{0}\right)}{\Gamma \left(1/2+2i{\tilde{\omega }}_{0}+r\right)\Gamma \left(1/2+2i{\tilde{\omega }}_{0}-r\right)},\;v_{-}=\frac{\Gamma \left(1+2i{\tilde{\omega }}_{0}\right)\Gamma \left(-2i{\tilde{\omega }}_{0}\right)}{\Gamma (1/2+r)\Gamma (1/2-r)}.$$

Using relation (36), we can simplify the expression for coefficient $v_{-}$:

(56) $$v_{-}=\frac{i\cos\left(\pi \sqrt{1/4-4{\tilde{\omega }}_{0}^{2}}\right)}{\sinh(2\pi {\tilde{\omega }}_{0})}.$$

The quantity $|v_{-}{|}^{2}$ increases with an increase of ${\tilde{\omega }}_{0}$. If ${\tilde{\omega }}_{0}=1/4$, then $|v_{-}{|}^{2}={\left[\sinh\left(\pi /2\right)\right]}^{-2}\approx 0.19$. In the adiabatic limit ${\tilde{\omega }}_{0}\gg 1$, we have $v_{-}\approx i\coth\left(2\pi {\tilde{\omega }}_{0}\right)$, i.e., $v_{-}\to i$ and $|v_{-}|\to 1$ when ${\tilde{\omega }}_{0}\to \infty$. In this limit, we have $r=2i{\tilde{\omega }}_{0}+O\left({\tilde{\omega }}_{0}^{-1}\right)$. Then, we can write

$$v_{+}\approx \frac{2i{\tilde{\omega }}_{0}{\left[\Gamma \left(2i{\tilde{\omega }}_{0}\right)\right]}^{2}}{\sqrt{\pi }\Gamma \left(1/2+4i{\tilde{\omega }}_{0}\right)}.$$

Using the asymptotic Stirling formula for the gamma function,

(57) $$\Gamma \left(z\right)\approx \sqrt{2\pi }\exp\left[(z-1/2)\ln(z)-z\right],\;\left|z\right|\gg 1.$$

we obtain the expression

(58) $$v_{+}\approx \sqrt{2}\;\exp\left[-4i{\tilde{\omega }}_{0}\ln\left(2\right)\right].$$

Consequently, $|v_{+}{|}^{2}-{\left|v_{-}\right|}^{2}=1$ and E(∞)/E(−∞)=3, in accordance with formula (38).

The asymptotic form (54) is similar to the general adiabatic solution (15). Using the definition (16) of the phase ϕ(t), we obtain the formula (remember that ω(t)=|tanh(κt/2)| in this section, as soon as function ω(t) is assumed to be non-negative in formula (15))

(59) $$\phi \left(t\right)=2{\tilde{\omega }}_{0}\ln\left[\cosh\left(\kappa t/2\right)\right]\mathrm{sign}\left(t\right).$$

If t→±∞, then $\phi \left(t\right)\approx \omega_{0}t-2{\tilde{\omega }}_{0}\ln\left(2\right)\mathrm{sign}\left(t\right)$. This means that, according to (15), the function $\omega_{0}^{-1/2}\exp\left(i\left[\omega_{0}t+2{\tilde{\omega }}_{0}\ln\left(2\right)\right]\right)$ at t→−∞ goes to the following superposition at t→∞:

$$\omega_{0}^{-1/2}\left\{u_{+}\exp\left(i\left[\omega_{0}t-2{\tilde{\omega }}_{0}\ln\left(2\right)\right]\right)+u_{-}\exp\left(i\left[-\omega_{0}t+2{\tilde{\omega }}_{0}\ln\left(2\right)\right]\right)\right\}.$$

Comparing this expression with (54), we conclude that

(60) $$u_{+}=v_{+}=\sqrt{2}\;\exp\left[-4i{\tilde{\omega }}_{0}\ln\left(2\right)\right],\;u_{-}=v_{-}=i.$$

We see that the phases of complex coefficients $u_{\pm }$ are sensitive to the rate of the adiabatic evolution through the term ${\tilde{\omega }}_{0}$. A strong consequence of this result is considered in the next section.

## 7. Double Adiabatic Passage of Frequency through Zero Value

What can happen if the frequency passes again through a zero value? Then, we have to make the transformation of function (15), using the superposition principle and two additional observations. First, the function $\omega^{-1/2}\exp\left(-i\phi \right)$ transforms as function $\varepsilon^{*}\left(t\right)$ after the frequency passes through a zero value. Second, applying the transformation rule (15) to the second transition, we must use the phase $\tilde{\phi }\left(t\right)$, where the integral over the frequency is taken from the second transition point $t_{**}$. Obviously,

(61) $$\phi \left(t\right)=\tilde{\phi }\left(t\right)+\Phi ,\;\Phi \left(t_{*},t_{**}\right)=\int_{t_{*}}^{t_{**}}\omega \left(z\right)dz.$$

We suppose that the transition rule through the second zero has the form

$${\left\{{\left[\omega \left(t\right)\right]}^{-1/2}e^{i\tilde{\phi }\left(t\right)}\right\}}_{t<t_{**}}\to {\left\{{\left[\omega \left(t\right)\right]}^{-1/2}\left[w_{+}e^{i\tilde{\phi }\left(t\right)}+w_{-}e^{-i\tilde{\phi }\left(t\right)}\right]\right\}}_{t>t_{**}},\;|w_{+}{|}^{2}-{\left|w_{-}\right|}^{2}=1.$$

Comparing the two forms of the solution ε(t) for $t>t_{**}$ (far enough from that point), we arrive at the equality (omitting the common term $\omega^{-1/2}$ and using the notation $U_{\pm }$ for the coefficients at $t>t_{**}$)

$$U_{+}e^{i\tilde{\phi }\left(t\right)}+U_{-}e^{-i\tilde{\phi }\left(t\right)}=u_{+}e^{i\Phi }\left[w_{+}e^{i\tilde{\phi }\left(t\right)}+w_{-}e^{-i\tilde{\phi }\left(t\right)}\right]+u_{-}e^{-i\Phi }\left[w_{+}^{*}e^{-i\tilde{\phi }\left(t\right)}+w_{-}^{*}e^{i\tilde{\phi }\left(t\right)}\right].$$

Hence,

(62) $$U_{+}=w_{+}u_{+}e^{i\Phi }+w_{-}^{*}u_{-}e^{-i\Phi },\;U_{-}=w_{-}u_{+}e^{i\Phi }+w_{+}^{*}u_{-}e^{-i\Phi }.$$

One can verify that the identity $|U_{+}{|}^{2}-{\left|U_{-}\right|}^{2}=1$ is fulfilled exactly. The adiabatic mean energy amplification factor after the second passage through a zero frequency equals

(63) $$\beta =1+2|U_{-}{|}^{2}=1+2\left[|w_{-}{|}^{2}|u_{+}{|}^{2}+|w_{+}{|}^{2}{\left|u_{-}\right|}^{2}+2Re\left(w_{+}w_{-}u_{+}u_{-}^{*}e^{2i\Phi }\right)\right].$$

In the adiabatic regime, Φ≫1. Moreover, this phase is very sensitive to the form of function ω(t) and the distance between the zero-point instances $t_{*}$ and $t_{**}$. In addition, coefficients $u_{\pm }$ and $w_{\pm }$ can be strongly phase-sensitive, as shown in Section 6. This means that it is practically impossible to predict the energy mean value after twice crossing the zero frequency (quite differently from the single crossing). The extremal values of β are as follows,

(64) $$\beta_{min}=1+2{\left(|w_{+}u_{-}\left|-\right|w_{-}u_{+}|\right)}^{2},\;\beta_{max}=1+2{\left(|w_{+}u_{-}\left|+\right|w_{-}u_{+}|\right)}^{2}.$$

In particular, if $w_{\pm }=u_{\pm }$, then $\beta_{min}=1$, meaning that, in principle, the mean energy can return to the initial value after the frequency passes through a zero value two times. On the other hand, $\beta_{max}=1+8{\left|u_{+}u_{-}\right|}^{2}$ under the same conditions. If n=2, $\beta_{max}=17$.

### Classical Illustrations

To illustrate the effects after single and double frequency crossings through a zero value, we considered the classical motion with the frequency $\omega^{2}\left(t\right)=\omega_{0}^{2}\sin^{2}\left[\pi t/\left(2\tau \right)\right]|$ for t≥=−τ and the initial conditions (5). Figure 8 shows the energy ratio *R* (6) at the instants T=t/τ=1 and T=3, for several values of parameter *G* close to G=1000. If T=1 (single crossing), the variations of parameter *G* result in shifts of the curves without changing the maximal, minimal and average values. On the other hand, the picture is totally different for T=3 (double crossing). In this case, Φ=4G/π. Hence, the variation ΔΦ=2π, when one can expect a similar behavior, corresponds to $\Delta G=\pi^{2}/2\approx 5$. On the other hand, a twice smaller variation ΔG≈2, yielding ΔΦ≈π, results in a totally different behavior, as one can see in the figure for T=3.

## 8. Energy Fluctuations

Figure 3, Figure 4 and Figure 8 show strong energy fluctuations (as functions of the initial phase) after the frequency passes through a zero value. These fluctuations can be characterized by the variance $\sigma_{E}=\langle E^{2}\rangle -{\langle E\rangle }^{2}$. Using the solutions (9) of the Heisenberg equations of motion, one can write $\sigma_{E}$ in terms of the fourth- and second-order moments of the canonical variables *x* and *p* and various products of functions ε(t), $\dot{\varepsilon }\left(t\right)$ and their complex-conjugated partners. The complete formula is rather cumbersome in the most general case. For this reason, we consider here the simplest case of the initial Fock quantum state |N〉. This special case is probably the most interesting, because the famous adiabatic theorem in quantum mechanics was proven by Born and Fock [23] exactly for the Fock states. In this special case (as well as for arbitrary diagonal mixtures of Fock states), the nonzero statistical moments are those containing *even powers* of each variable, *x* or *p*. After some algebra, one can obtain the following formula (using the dimensionless variables, assuming $\hbar =m=\omega_{0}=1$, so that $\langle x^{4}\rangle =\langle p^{4}\rangle$):

(65) $$16{\langle E^{2}\rangle }_{t}=2{\langle x^{4}\rangle }_{-\tau }\left(A^{2}+B^{2}\right)+{\langle x^{2}p^{2}+p^{2}x^{2}\rangle }_{-\tau }\left(A^{2}-B^{2}\right)+{\langle {\left(xp+px\right)}^{2}\rangle }_{-\tau }C^{2},$$

where

A(t)=ω2(t)|ε(t)|2+|ε˙(t)|2,B(t)=Reω2(t)ε2(t)+ε˙2(t),C(t)=Imω2(t)ε2(t)+ε˙2(t).

In the adiabatic regime (15), we have

(66) A=2ω(t)u+|2+|u−|2,B=4ω(t)Reu+u−,C=4ω(t)Imu+u−.

For the initial Fock state |N〉, we have

$${\langle x^{4}\rangle }_{-\tau }=\frac{3}{4}\left(2N^{2}+2N+1\right),\;{\langle x^{2}p^{2}+p^{2}x^{2}\rangle }_{-\tau }=\frac{1}{2}\left(2N^{2}+2N-1\right),$$

$${\langle {\left(xp+px\right)}^{2}\rangle }_{-\tau }=2\left(N^{2}+N+1\right).$$

Hence,

$${\langle E^{2}\rangle }_{t}/\omega^{2}\left(t\right)={\left(u_{+}{|}^{2}+{\left|u_{-}\right|}^{2}\right)}^{2}{\left(N+1/2\right)}^{2}+2{\left|u_{+}u_{-}\right|}^{2}\left(N^{2}+N+1\right).$$

Remembering that the mean energy equals ${\langle E\rangle }_{t}=\omega \left(t\right)\left(u_{+}{|}^{2}+{\left|u_{-}\right|}^{2}\right)\left(N+1/2\right)$, we arrive at the unexpectedly simple formula for the energy variance:

(67) $$\sigma_{E}\left(t\right)=2\omega^{2}\left(t\right)|u_{+}u_{-}{|}^{2}\left(N^{2}+N+1\right),\;\frac{\sigma_{E}\left(t\right)}{{\langle E\rangle }_{t}^{2}}=2{\left|u_{+}u_{-}\right|}^{2}\frac{N^{2}+N+1}{N^{2}+N+1/4}.$$

In the absence of zero-frequency values we have $u_{-}=0$. In this case, $\sigma_{E}\left(t\right)\equiv 0$, in accordance with the Born–Fock theorem. However, this theorem is broken when the frequency passes through a zero value. For example, for the initial vacuum state (N=0) and the power index n=2 of the single frequency transition through a zero value, we obtain $\sigma_{E}\left(t\right)/{\langle E\rangle }_{t}^{2}=16$. This ratio can be four times smaller if N≫1.

In quantum optics, fluctuations are frequently characterized by the *Mandel factor* [24]

(68) $$Q=\left(\langle {\hat{n}}^{2}\rangle -{\langle \hat{n}\rangle }^{2}\right)/\langle \hat{n}\rangle -1,\;\hat{n}\equiv \hat{E}/\left(\hbar \omega \right)-1/2.$$

Then, for the initial Fock state |N〉 (having Q=−1, which means the so called sub-Poissonian statistics), we obtain the following instantaneous values:

$$\hat{n}=N+2{\left|u_{-}\right|}^{2}\left(N+1/2\right),\;\langle {\hat{n}}^{2}\rangle -{\langle \hat{n}\rangle }^{2}=\sigma_{E}/{\left(\hbar \omega \right)}^{2}.$$

Consequently,

(69) $$Q=\frac{|u_{-}{|}^{2}\left(1+2|u_{-}{|}^{2}\right)+N\left(2|u_{-}{|}^{4}-1\right)+2N^{2}{\left|u_{+}u_{-}\right|}^{2}}{N+2|u_{-}{|}^{2}\left(N+1/2\right)}.$$

In particular, for $|u_{-}{|}^{2}=1$, we have

(70) $$Q=\frac{3+N+4N^{2}}{3N+1}.$$

This means that the statistics become super-Poissonian after the frequency passage through a zero value. However, the super-Poissonianity is not very strong, because $Q\approx \left(4/3\right)N\approx \left(4/9\right)\langle \hat{n}\rangle$ for N≫1, whereas $Q=\langle \hat{n}\rangle$ for the “strongly super-Poissonian” thermal states.

## 9. Evolution of the Fock States

What happens with the initial Fock state |N〉 when the frequency passes through a zero value? Obviously, it cannot survive, as soon as the mean energy and especially the energy variance increase substantially. This means that the initial Fock state becomes a superposition of many Fock states. However, what is the width of the new distribution? Is it concentrated near some distinguished states, or it is very wide and almost uniform, especially when N≫1? To answer these questions, one can use general results concerning the quantum harmonic oscillator with a time-dependent frequency [11,12,13,14] (the details can be found, e.g., in the review [25]). Recall that the Fock states |N〉 are eigenstates of the operator ${\hat{a}}^{†}\hat{a}$, where $\hat{a}$ and ${\hat{a}}^{†}$ are standard annihilation and creation operators. When the frequency varies with time, operators $\hat{a}$ and ${\hat{a}}^{†}$ become new operators, $\hat{A}$ and ${\hat{A}}^{†}$, which are quantum integrals of motion. The time-dependent state ${|N\rangle }_{t}$ remains the eigenstate of operator ${\hat{A}}^{†}\hat{A}$. As soon as operators $\hat{A}$ and ${\hat{A}}^{†}$ maintain their *linear* form with respect to operators $\hat{x}$ and $\hat{p}$, the wave function of state ${|N\rangle }_{t}$ maintains its functional form as the product of some Gaussian exponential by the Hermite polynomial. The explicit form, found in [12,14] (see also [18]), is as follows (in dimensionless units with ℏ=m=1),

(71) $${\langle x|N\rangle }_{t}={\left(N!\;\varepsilon \sqrt{\pi }\right)}^{-1/2}{\left(\frac{\varepsilon^{*}}{2\varepsilon }\right)}^{N/2}\exp\left(\frac{i\dot{\varepsilon }}{2\varepsilon }x^{2}\right)H_{N}\left(\frac{x}{\left|\varepsilon \right|}\right),$$

where ε(t) is the solution to Equation (2) satisfying conditions (7) and (8). Transition probabilities ${|\langle M|N\rangle }_{t}{|}^{2}$ between the instantaneous Fock state |M〉 (when $\varepsilon \left(t\right)=\omega^{-1/2}\exp\left(i\omega t\right)$) and exact time-dependent state ${|N\rangle }_{t}$ were calculated in different forms in [11,12,13,14]. In the generalized adiabatic regime (15), the results of [12,14] can be written in the form (symmetric with respect to *M* and *N*)

(72) $${|\langle M|N\rangle }_{t}{|}^{2}=\frac{N_{<}!}{N_{>}!}{\left|u_{+}\right|}^{-1}{\left[P_{(M+N)/2}^{|M-N|/2}\left(|u_{+}{|}^{-1}\right)\right]}^{2},$$

where $P_{j}^{k}\left(z\right)$ is the associated Legendre polynomial, $N_{<}=min\left(M,N\right)$, $N_{>}=max\left(M,N\right)$. Formula (72) holds provided |M−N|/2 is an integer; otherwise, the probability equals zero. Note that the probabilities do not depend on time, as soon as the adiabatic solution (15) is valid.

In some cases, it can be convenient to use the expression of the associated Legendre polynomials in terms of the Gauss hypergeometric function,

(73) $$P_{n}^{m}\left(x\right)=\frac{{\left(-1\right)}^{m}\left(n+m\right)!}{2^{m}\left(n-m\right)!m!}{\left(1-x^{2}\right)}^{m/2}F\left(m-n,m+n+1;m+1;\frac{1-x}{2}\right).$$

Then,

(74) $${|\langle M|N\rangle }_{t}{|}^{2}=\frac{2\left(N_{>}\right)!{\left|u_{-}\right|}^{\left|M-N\right|}}{\left(N_{<}\right)!{\left[\left(\frac{\left|M-N\right|}{2}\right)!\right]}^{2}{\left|2u_{+}\right|}^{|M-N|+1}}{\left[F\left(-N_{<}\;,N_{>}+1;\frac{\left|M-N\right|}{2}+1;\frac{|u_{+}|-1}{2|u_{+}|}\right)\right]}^{2}.$$

In the case of a single frequency crossing through a zero value, this formula can also be written as

(75) $${|\langle M|N\rangle }_{t}{|}^{2}=\frac{\left(N_{>}\right)!\sin\left(\nu \pi \right){\left[\cos\left(\nu \pi \right)\right]}^{\left|M-N\right|}}{\left(N_{<}\right)!{\left[\left(\frac{\left|M-N\right|}{2}\right)!\right]}^{2}2^{\left|M-N\right|}}{\left[F\left(-N_{<}\;,N_{>}+1;\frac{\left|M-N\right|}{2}+1;\sin^{2}\left[\left(1-2\nu \right)\pi /4\right]\right)\right]}^{2}.$$

Among different special cases, we bring here two formulas:

(76) $${|\langle 2K|0\rangle }_{t}{|}^{2}=\frac{\left(2K-1\right)!!|u_{-}{|}^{2K}}{\left(2K\right)!!|u_{+}{|}^{2K+1}}=\frac{(2K-1)!!}{(2K)!!}\sin\left(\nu \pi \right){\left[\cos\left(\nu \pi \right)\right]}^{2K},$$

(77) $${|\langle N|N\rangle }_{t}{|}^{2}={\left|u_{+}\right|}^{-1}{\left[P_{N}\left(|u_{+}{|}^{-1}\right)\right]}^{2}=\sin\left(\nu \pi \right){\left[P_{N}\left(\sin(\nu \pi )\right)\right]}^{2},$$

where $P_{N}\left(z\right)\equiv P_{N}^{0}\left(z\right)$ is the usual Legendre polynomial. The first equalities in Equations (76) and (77) hold in the most general adiabatic case (including multiple frequency crossings through a zero value), whereas the second equalities are valid for the single crossing. The distribution (76) (describing the evolution of the initial ground state) decreases monotonously as function of parameter *K*. However, the situation is totally different for other initial Fock states, especially when N≫1.

The survival probabilities $p_{s}\left(N\right)\equiv {\left|{\langle N|N\rangle }_{t}\right|}^{2}$ rapidly diminish with the quantum number *N*. For example, if n=2 (ν=1/4), we find the following surviving probabilities after the single frequency zero-crossing (when the mean energy triplicates):

$$p_{s}\left(0\right)=\frac{1}{\sqrt{2}},\;p_{s}\left(1\right)=\frac{1}{2\sqrt{2}},\;p_{s}\left(2\right)=\frac{1}{16\sqrt{2}},\;p_{s}\left(3\right)=\frac{1}{32\sqrt{2}},\ldots$$

This means that the initial Fock state |N〉 becomes a superposition of a large number of different Fock states |M〉, see Figure 9. It is impressive that the probability of transition N→3N is very small, whereas the probability $p_{N}\left(M\geq 3N\right)$ is about 50%. In addition, the distribution of probabilities $p_{N}\left(M\right)$ with M<3N looks rather irregular, whereas some regular picture is observed for M>3N. Unfortunately, we did not succeed to find an analytic approximation for this regular picture.

## 10. Squeezing Evolution

If the frequency ω does not depend on time, the evolution of the coordinate variance is given by the formula

(78) $$\sigma_{x}\left(t\right)=\sigma_{x}\left(0\right)\cos^{2}\left(\omega t\right)+\frac{\sigma_{p}\left(0\right)}{{\left(m\omega \right)}^{2}}\sin^{2}\left(\omega t\right)+\frac{\sigma_{xp}\left(0\right)}{\left(m\omega \right)}\sin\left(2\omega t\right).$$

Minimizing this expression over time, one can write the minimal value $\sigma_{min}$ as (similar formulas were obtained, e.g., in [26,27,28])

$$\sigma_{min}={\left(m\omega^{2}\right)}^{-1}\left[E-\sqrt{E^{2}-\omega^{2}D}\right],$$

where

$$E=\sigma_{p}/\left(2m\right)+m\omega^{2}\sigma_{x}/2,\;D=\sigma_{x}\sigma_{p}-\sigma_{xp}^{2}.$$

The quantity *D* is the simplest example of *quantum universal invariants* [29], which do not depend on time (although they depend on the initial state) for arbitrary quadratic Hamiltonians. On the other hand, $D\geq \hbar^{2}/4$ for any (normalizable) quantum state due to the Schrödinger–Robertson uncertainty relation. The energy of quantum fluctuations *E* satisfies the inequality E≥ℏω/2. Therefore, it is convenient to use two dimensionless parameters, λ≥1 and γ≥1, according to the relations E=λℏω/2 and $D=\gamma^{2}\hbar^{2}/4$. Then, normalizing the minimal value $\sigma_{min}$ by the variance in the vacuum state $\sigma_{vac}=\hbar /\left(2m\omega \right)$, one can obtain the following formula for the *invariant squeezing coefficient*$s=\sigma_{min}/\sigma_{vac}$:

(79) $$s=\lambda -\sqrt{\lambda^{2}-\gamma^{2}}=\frac{\gamma^{2}}{\lambda +\sqrt{\lambda^{2}-\gamma^{2}}}.$$

For the states satisfying the initial conditions (21), we have γ=λ and s=λ≥1. Moreover, parameter λ maintains it initial value in the standard adiabatic case (14). However, if the frequency passes through a zero value, in the new adiabatic regime (18), λ goes to βλ, while γ maintains its initial value. Hence, the new squeezing coefficient equals

(80) $$s=\frac{\lambda }{\beta +\sqrt{\beta^{2}-1}}.$$

Hence, the initial vacuum state becomes squeezed when the frequency passes adiabatically through a zero value. Using Equation (38), we obtain the following value of the squeezing coefficient after a single passage through zero:

(81) $$s=\lambda \tan^{2}\left(\nu \pi /2\right).$$

In particular, s≈0.17λ for ν=1/4 (i.e., n=2 and β=3), so the Fock states |N〉 become squeezed for N≤2 (when λ≤5) in this special case.

## 11. Conclusions

The first main result of the paper is the discovery of the existence of the generalized adiabatic invariant in the form of Equation (18). In the most general case, the adiabatic proportionality coefficient in this equation depends on the initial state. This dependence disappears after averaging over parameters of families of initial states with the same energy (in particular, such averaging happens automatically for the initial vacuum, Fock and thermal states). Then, universal relations (45) exist, *provided the frequency passes through zero only once*. In the cases of multiple frequency passages through zero, the energy adiabatic coefficients become sensitive to the additional parameter, the phase Φ, according to Equation (63). As a consequence, the adiabatic behavior after many crossings through a zero frequency value can be quasi-chaotic. Under specific conditions, the mean energy can return to the initial value after a double frequency passage through zero. The original Born–Fock adiabatic theorem is broken after the frequency passes through a zero value. Although the functional shape of the wave function of the initial Fock state is preserved in the form of the product of a Gaussian exponential by the Hermite polynomial, the arguments of this form are not determined totally by the instantaneous frequency. However, the probability distribution over the instantaneous Fock states, determined by the adiabatic coefficients $|u_{\pm }|$, according to Equation (72), does not depend on time, as soon as the adiabatic regime is justified. This statement can be considered as the generalized Born–Fock theorem; it is the second main result of the paper. Note that the time-independent probability distributions can be different after each frequency passage through zero.

In view of the mean energy amplification (e.g., triplication in the most natural case of a linear frequency dependence near the zero point), any initial state becomes significantly deformed. For example, coherent states (which possess the same quadrature variances as the vacuum state) will be transformed into squeezed states. The same can be said about initial thermal states: they will become Gaussian mixed states with unequal quadrature variances (and squeezed under certain conditions), maintaining the initial value of the quantum purity.

## Figures and Tables

**Figure 1:**
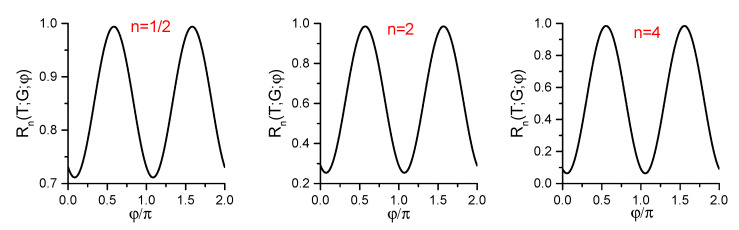
The dimensionless energy of a classical particle at the dimensionless instant T=−1/2 in the nonadiabatic regime (G=1).

**Figure 2:**
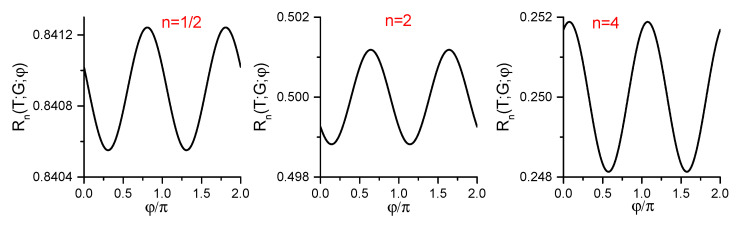
The dimensionless energy of a classical particle at the dimensionless instant T=−1/2 in the adiabatic regime (G=1000).

**Figure 3:**
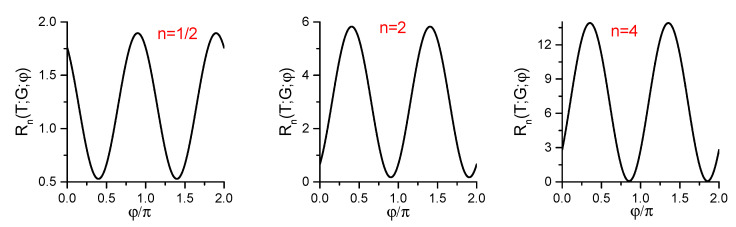
The dimensionless energy of a classical particle at the dimensionless instant T=1 in the case of slow evolution (G=1000), for the profile $f\left(T\right)={\left|T\right|}^{n}$.

**Figure 4:**
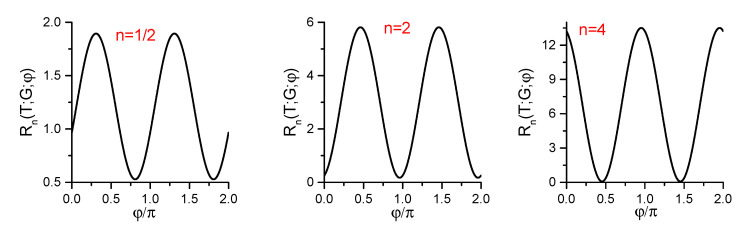
The dimensionless energy of a classical particle at the dimensionless instant T=1 in the case of slow evolution (G=1000) for the profile $f\left(T\right)={\left|\tanh\left(aT\right)/\tanh\left(-a\right)\right|}^{n}$a=5.

**Figure 5:**
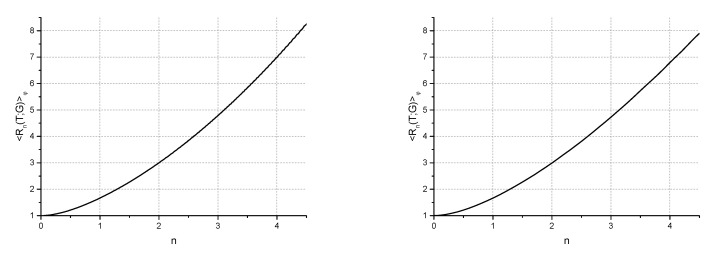
The dimensionless energy of a classical particle at the dimensionless instant T=1 in the case of slow evolution (G=1000), averaged over the initial phase φ, as function of index *n*. Left: for the profile $f\left(T\right)={\left|T\right|}^{n}$. Right: for the profile $f\left(T\right)={\left|\tanh\left(aT\right)/\tanh\left(-a\right)\right|}^{n}$, a=5.

**Figure 6:**
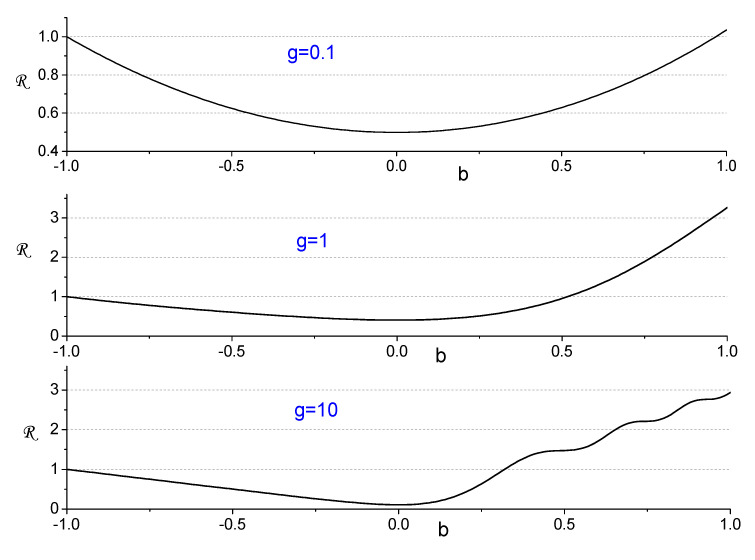
The function R(b) for g=0.1,1.0,10 and ν=1/4.

**Figure 7:**
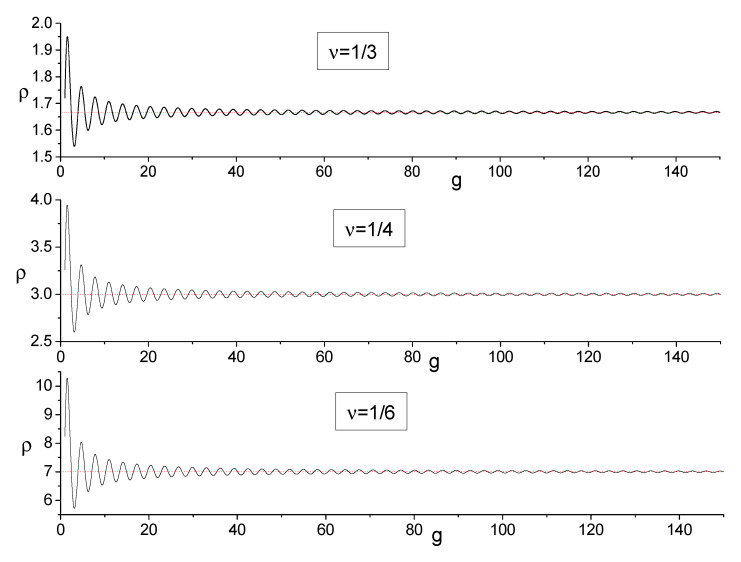
The function ρ(g) (39) for g>1 and ν=1/3,1/4,1/6.

**Figure 8:**
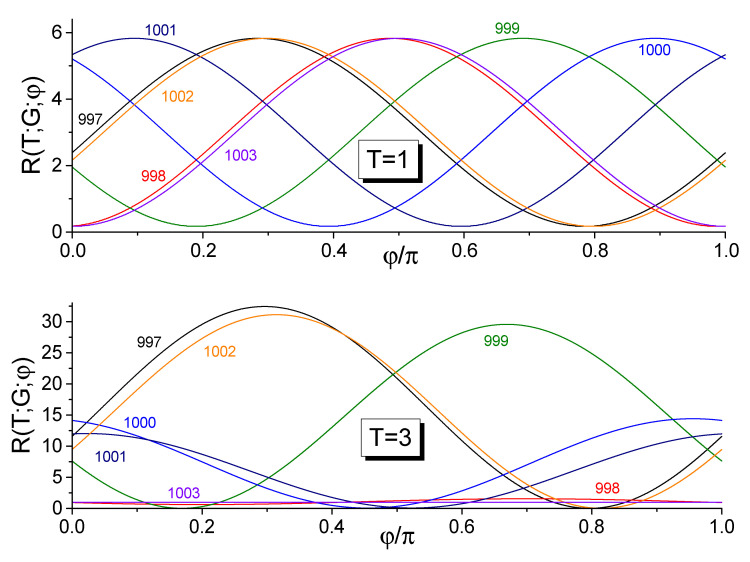
The dimensionless energy of a classical particle at the dimensionless instants T=1 (**top**) and T=3 (**bottom**) for several different values of the adiabatic parameter $G=\omega_{0}\tau$, shown near the respective lines. The frequency profile is $\omega^{2}\left(t\right)=\omega_{0}^{2}\sin^{2}\left[\pi t/\left(2\tau \right)\right]|$.

**Figure 9:**
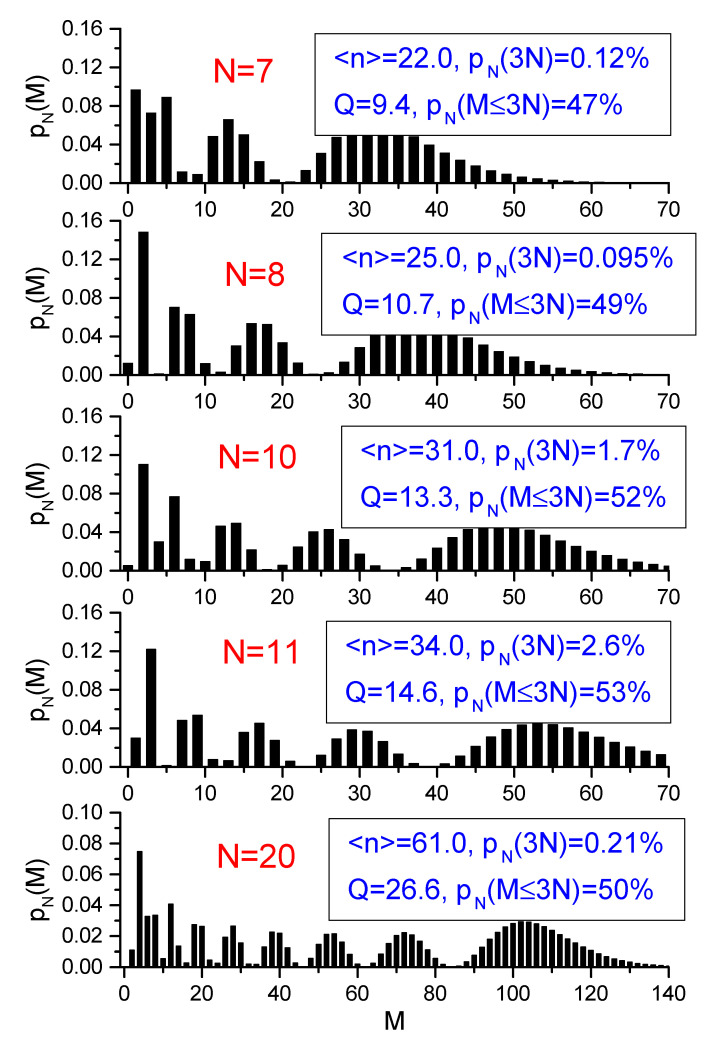
The probability p(M) (given by Equation (74)) of finding the initial Fock state |N〉 in the Fock state |M〉 after the frequency slowly passes through a zero value, in the case of $|u_{-}|=1$ and $|u_{+}|=\sqrt{2}$.

## Data Availability

Not applicable.
